# High relevance of invasive fungal disease in chronic liver transplant failure: a comprehensive cross-sectional study

**DOI:** 10.1007/s00428-025-04050-4

**Published:** 2025-02-18

**Authors:** Aleksandra V. Puzalkova, Katharina Hofmann, Tilman Pfeffer, Isabel M. Klein, Arianeb Mehrabi, Uta Merle, Albrecht Stenzinger, Roland Penzel, Christa Flechtenmacher, Peter Schirmacher

**Affiliations:** 1 Institute of Pathology, Heidelberg University Hospital, Heidelberg, Germany; 2 Tissue Bank of the German Center for Infection Research (DZIF), Institute of Pathology, Heidelberg University Hospital, Heidelberg, Germany; 3 Department of General, Visceral and Transplantation Surgery, Heidelberg University Hospital, Heidelberg, Germany; 4 Department of Gastroenterology, Infectious Diseases and Intoxication, Internal Medicine IV, Heidelberg University Hospital, Heidelberg, Germany

**Keywords:** Mycosis, Invasive fungal infection, Liver transplantation, Chronic transplant failure

## Abstract

Invasive fungal infection (IFI) is a severe complication in organ transplant patients and a major diagnostic and therapeutic challenge. The aim of this comprehensive retrospective study was the characterization of IFI in context of chronic liver transplant failure regarding prevalence, morphological changes, and inducing fungal species. All explanted liver transplants due to chronic transplant failure from Heidelberg University Hospital were extensively reexamined for the presence of mycotic infection. Special stains were performed to uncover severity and associated conditions of IFI and fungal species were identified on the molecular level. Light-microscopic examination revealed fungal infection in 41 (27.5%) of 149 explanted livers with chronic transplant failure, with 68% representing newly specified cases compared to primary reports. We could show an increase of the proportion of mycotic infections during the investigated time period (1991–2021) as well as a shorter transplant survival when compared to the non-affected organs. Typically, large bile ducts were affected, accompanied by acute inflammation with frequent abscess and bile concrement formation. In 35 of 41 cases, the identification of the fungal species was achieved, revealing *Candida albicans* as prominent species (74.3%). In three autopsy livers of patients that died after liver transplantation of septic multiorgan failure, *Candida* spp. could be identified. Our data show the underestimated prevalence and high diagnostic and clinical relevance of mycotic infection in chronic liver transplant failure. Adapting diagnostic procedures, molecular pathological analyses, and therapeutic strategies is relevant to identify and prevent chronic transplant organ failure caused by IFIs.

**Supplementary Information:**

The online version contains supplementary material available at 10.1007/s00428-025-04050-4.

## Introduction

The outcomes of liver transplantation have been steadily improved, leading to a 1-year patient survival of 86% in Europe [1]. Although early mortality rates have been drastically reduced, the rates of patient death and late graft loss remained stable during the past two decades, presenting a significant challenge in long-term transplant management. The causes of chronic liver allograft dysfunction are multiple, including late cellular rejection (> 90 days post transplantation), disease recurrence, and ischemic and biliary complications, leading to transplant loss and deaths [2, 3]. According to the European Liver Transplant Registry, the prevalence of liver retransplantation accounts for 5% of the registry cases [1, 3].

Invasive fungal infections (IFIs) are a widespread public health issue, not infrequently with fatal outcomes. Recent investigations estimated that more than 6.5 million people suffer from IFI annually, with approximately 3.8 million deaths [4]. Especially immunocompromised patients such as transplant recipients have a higher predisposition for IFIs. It is therefore a complication of high clinical relevance after liver transplantation and known to significantly contribute to morbidity, mortality, and graft loss [5–8]. Reported incidences of IFI in liver transplant recipients are highly variable, ranging between 2 and 42%, increase with time after transplantation [5, 6, 9, 10], and show mortality rates between 25 and 80% [6, 7, 9, 11, 12]. The reported species spectrum is heterogenous. *Candida* appear to be the major cause of IFI in post-liver-transplants [5, 6, 8, 9, 13, 14]. Infections by this genus are mainly intraabdominal, like peritonitis and abdominal abscesses, followed by biliary tract infections. *Aspergillus* mostly infects the airways or sinuses [10]. Different risk factors have been reported to be associated with IFIs in liver transplant recipients, e.g., preexisting *Candida* colonization, repetitive laparotomy, retransplantation, renal replacement therapy/renal dysfunction, choledochojejunostomy, broad-spectrum antibiotic therapy, diabetes, and cytomegalovirus infection [6–9].

In the clinical setting, the diagnosis of IFI is culture-based using samples obtained from an otherwise sterile site or tissue-based by histopathology [5, 8, 9]. Sensitivities of culture-based methods are restricted and vary depending on fungal load and type of infection [9]. In case of *Candida*, a sensitivity of 60–80% is reported for blood cultures, limited by the rapid clearance of yeasts from the blood stream [5, 6, 9]. This is why the guideline for the diagnosis and management of *Candida* infections by the European Society of Clinical Microbiology and Infectious Diseases (ESCMID) recommends the additional use of alternative detection strategies [15].

Due to the life-threatening outcomes of IFI, antifungal prophylaxis for recipients of solid organ transplants has been established, especially due to diagnostic limitations as well as a poor treatment outcome in case of delayed therapy. Prophylaxis can be performed as an universal (all patients), targeted (only high-risk patients), or preemptive strategy (patients with known fungal colonization) [6]. Due to adverse events, guidelines differ in recommending a targeted prophylaxis for high-risk patients or a universal prophylaxis. The EASL guideline recommends a universal prophylaxis against *Candida* species during the first months after liver transplantation. At present, fluconazole is the most commonly used antifungal agent [16]. Despite prophylaxis, breakthrough IFI occurs with a reported frequency of 5% of liver transplant patients [17].

As IFIs of solid organ transplants are a relevant diagnostic and therapeutic challenge, we assessed prevalence and significance of invasive mycosis in chronic liver transplant failure. A comprehensive analysis of the disease pattern and morphologic changes of all explanted transplant livers at University Hospital Heidelberg was performed based on histopathological analyses to enable insights into the mechanisms and outcomes of IFI. Additionally, molecular classification of pathogens on the species level was performed. Our data provide novel insights into impact and mechanisms of IFI in chronic liver transplant failure that affect diagnostic assessment and therapeutic strategies.

## Materials and methods

### Patient cohort

All 157 patients with explanted liver transplants representing 192 explanted transplant livers between 1991 and 2021 at the University Hospital Heidelberg were extracted from the transplant registry of the Department of General and Transplant Surgery. Primary inclusion criterium was posttransplant survival ≥ 90 days to represent chronic transplant failure and eliminate cases with acute transplant dysfunction. Subsequently, all cases were matched to explanted liver tissue samples present in the archive of the Institute of Pathology Heidelberg (IPH). Additionally, three autopsy cases of the IPH representing liver transplant patients that died of septic multiorgan failure meeting the criteria were included in the study cohort. By this, 149 cases of chronic transplant liver failure in 136 patients were subjected to systematic histopathological analysis. For 11 of these patients, a second re-transplantation was included, and one patient underwent three re-transplantations. All histological reports were evaluated for the characterization of fungal infection. Figure S1 shows a detailed description of the cohort recruitment. The study was designed and performed according to the STROBE statement for cross sectional reporting guidelines [18]. Research was performed under the ethics approval S-206/2005 by the Ethics Committee Heidelberg and according to the Declaration of Helsinki 2013.

### Histopathological analyses

In all cases, all liver FFPE samples (min. four samples per explanted liver) were analyzed using HE, GMS, and PAS stains for the presence of mycosis. Mycotic infection of an explanted transplant liver was defined if at least one tissue sample showed definitive histological evidence for fungal species as well as signs of inflammation. Furthermore, the localization of the infection, morphological changes, and the type of inflammation (acute/acute on chronic) were characterized. Additionally, all available liver biopsies in the timeframe of 12 months prior to explantation were reevaluated for fungal infection. All histological analyses were performed by two board-certified pathologists (C. F., P. S.).

**Fig. 1:**
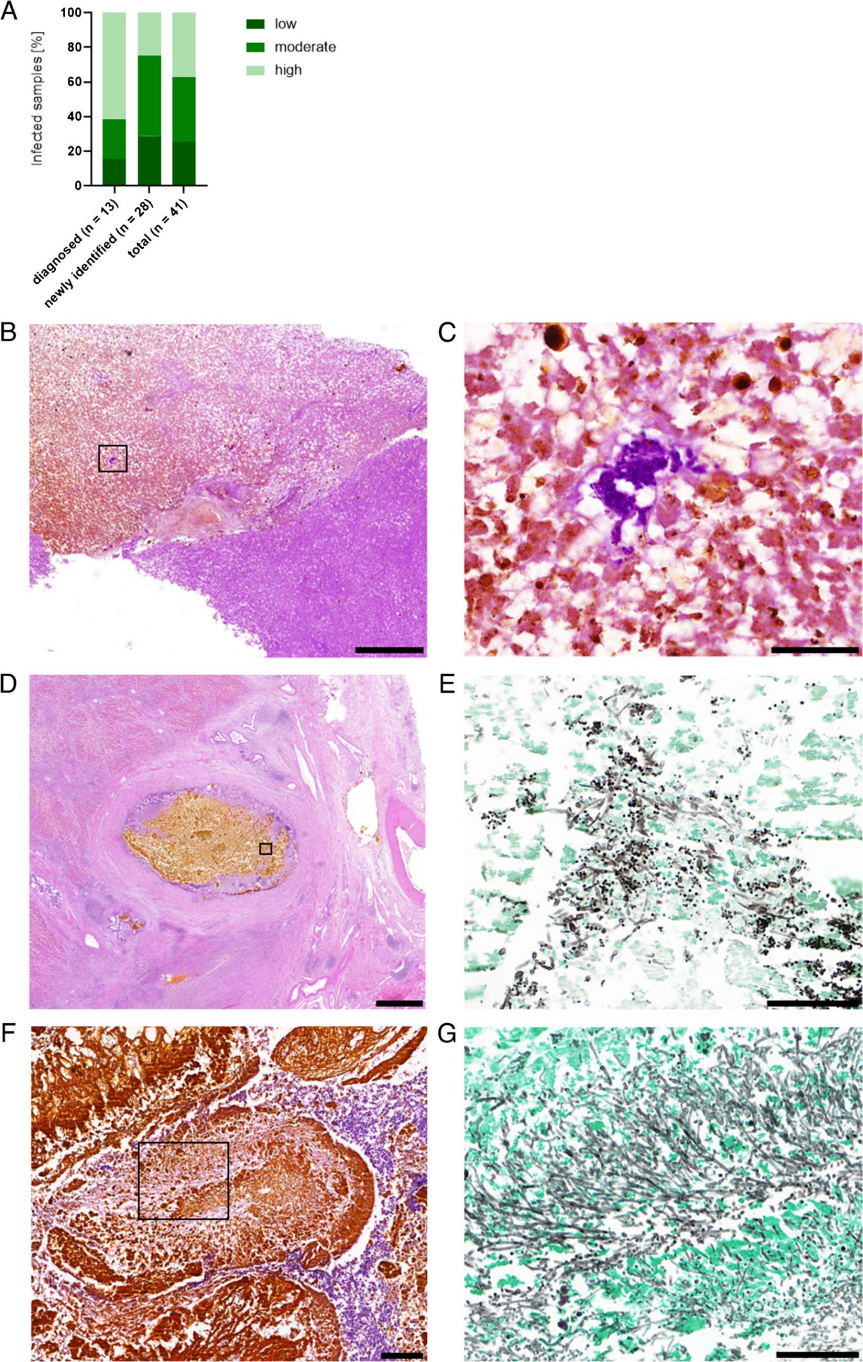
Classification of the severity of IFI. **A** The 41 identified IFI cases were classified in low (dark green), moderate (medium green), and high (bright green) loads of fungal structures and amount of infected tissue. **B** Exemplary microscopic pictures of biliary necrosis including a small inclusion with low fungal load (HE stain), with **C** showing a detail of **B** with PAS stain. **D** A large bile duct with biliary concrement and a moderate fungal load (HE stain), with **E** showing a detail of **D** with GMS stain. **F** A large bile duct with biliary abscess and high fungal load (HE stain), with **G** showing a detail of **F** in GMS stain. The used parts for the details are marked with a black square. Scale bar: A, C = 1000 µm; B, D, F = 100 µm; E = 200 µm

An acute inflammation was classified in case of presence of neutrophil-rich infiltrations in and around the affected hepatic structures, i.e., biliary epithelium and lumina, or vascular wall and lumina in the cases with affected blood vessels. For an acute-on-chronic inflammation, additional significant infection-associated fibrosis had to be present [19]. The cases were further classified semi-quantitatively in low, moderate, and high histologically detectable fungal load, based on the proportion of slides with detectable fungi in each case, the amount of visible fungal structures such as spores and/or hyphae on the affected slides, and the involved hepatic structures. For example, a case with only a small proportion of affected slides, consisting of singular biliary tract involvement with few fungal structures, was considered as “low fungal load,” whereas a case with multilocular mycotic biliary abscesses, detectable on a large proportion of slides and showing high number of fungal species, would be considered as “high fungal load” (see exemplary pictures in Fig. 1C, E, G).

### Molecular fungal analyses

FFPE samples with evidence for mycosis were further investigated using molecular pathologic methods to specify the fungal pathogen. For this purpose, areas with fungal structures were marked by an experienced pathologist on an H&E-stained slide, and the corresponding tissue areas were manually microdissected from subsequent unstained slides. Extraction of genomic DNA was performed after Proteinase K digestion on a fully automated Maxwell 16 purification system using the LEV DNA FFPE Purification Kit (Promega, Madison, USA). The DNA content was measured fluorometrically using a Qubit dsDNA HS assay (Life Technologies Carlsbad, USA). Subsequently, genomic DNA was amplified with the VisionArray FUNGI PreCise Master Mix 1.0 according to the manufacturer’s instructions (Zytomed Systems, Berlin, Germany). The identification of fungal species was performed with the VisionArray® FUNGI Chip 2.0 as described in the manufacturer’s specifications (Zytomed Systems). This assay is based on DNA/DNA-hybridization of the PCR products to DNA capture sequences of 30 different fungal species. The visualization of specifically bound PCR products takes place by staining the conjugated streptavidin-peroxidase with tetramethylbenzidine.

### Statistical analyses

**Fig. 2:**
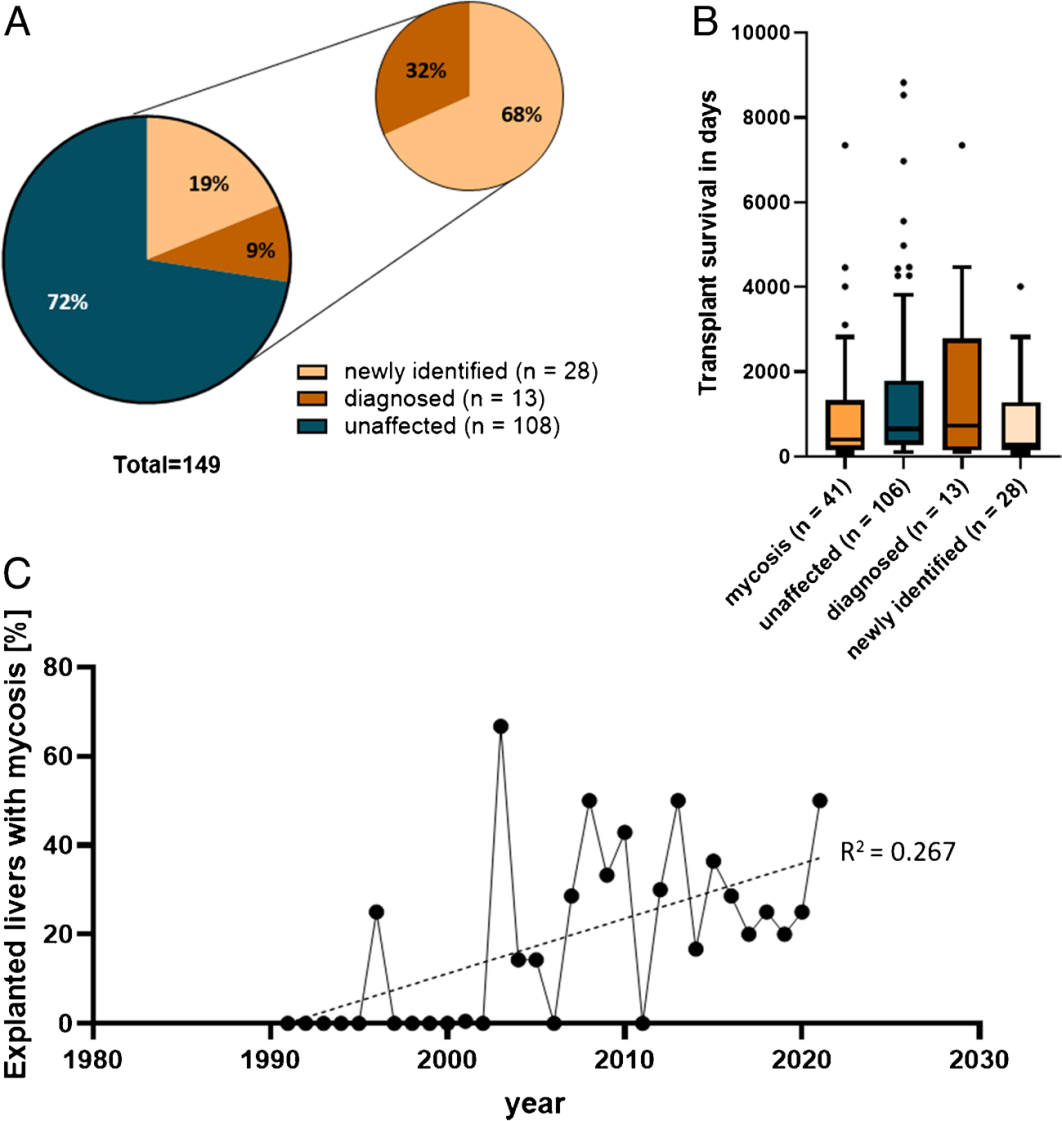
Histopathological investigations led to a discovery of unknown cases of IFI. The collective of 149 explanted transplant livers were investigated for IFIs. **A** Cases were classified in unaffected cases without mycoses (blue), previously diagnosed mycoses (dark orange), and mycoses that were newly identified by histopathological investigations (bright orange). **B** Transplant survival of livers with identified mycoses (orange) and without mycoses (blue) in days. **C** Percentage of liver explants with histopathologically identified mycosis between 1991 and 2021. Dotted line: linear regression

Testing for statistically significant differences in mean values was performed using a One-way ANOVA (Fig. 2B). *R*^2^ value of Fig. 2C was calculated by a simple linear regression. All statistical analyses were performed using the GraphPad Prism9 software version 9.0.2 (San Diego, CA, USA).

## Results

### Tissue-based identification of fungal transplant infection

By histopathological analyses, the presence of mycosis in 41 out of 149 (27.5%) explanted transplant livers was revealed. In 13 (31.7%) of these cases, a fungal infection was previously explicitly pathologically diagnosed, whereas the remaining 28 cases (68.3%) represented IFIs that were newly identified in addition to the original diagnostic report (Fig. 2A). From the 12 patients that underwent a second or third transplantation, 3 patients showed mycoses in both transplants, 2 of them in the liver transplant obtained from the autopsy. When reevaluating hepatobiliary specimen taken in 12 months prior to explantation for IFI-positive cases, fungal infection was found solely in one case of a preceding bile duct segment resection. All other tissues were free of fungal infection. In addition, the clinical records of all patients were systematically searched whether clinico-serologically a fungal infection was known or at least suspected prior to the retransplantation. Of the 41 cases, the existence of a fungal infection was known or suspected in 21 cases (51.2%), and in 15 cases (36.6%), this fungal infection was clinically related to the liver.

Regarding the age of transplant patients at the time of liver transplant explantation, only slight differences between the cases with (Ø 50 years) and without mycosis (Ø 47 years) were found. Generally, the whole cohort was composed of 71.8% men and 28.2% women. However, the mycosis positive samples showed a slight overrepresentation of the female gender (34.1%) compared to the group without IFIs (25.9%). For the duration of transplant survival, unaffected transplants had longer survival times (Ø 1378 days) than the ones with mycosis (Ø 1033 days). From those, the 28 cases that were previously diagnosed showed a longer transplant survival (Ø 1682 days) compared to the ones that were identified by the histopathological analyses (Ø 732 days) (Table 1, Fig. 2B).

**Table 1:** Age, sex, and transplant survival of the study cohort

		Mycoses			Unaffected (*n* = 108)	Total cohort (*n* = 149)
		Diagnosed (*n* = 13)	Newly identified (*n* = 28)	Total mycoses (*n* = 41)		
Age	Mean	52	49	50	47	48
	Median	52	49.5	50	50	50
	Min	36	23	23	4	4
	Max	63	64	64	68	68
Sex	Male	8	19	27	80	107
	Female	5	9	14	28	56
	% Female	38.5	32.1	34.1	25.9	28.2
	% Male	61.5	67.9	65.9	74.1	71.8
Transplant survival (days)	Mean	1682	732	1033	1378	1282
	Median	720	273	397	659	508
	Min	97	92	92	99	92
	Max	7345	4012	7345	8820	8820

For further analyses of changes of the prevalence of mycoses in the liver explants, the percentage of mycosis within all liver explants was compared over the decades. Figure 2C shows a tendency for an increase of mycoses in liver explants between 1990 and 2021. Between 1990 and 2000, 7.7% of explanted livers showed mycotic infections, 31.0% from 2001 to 2010, and 27.8% from 2011 to 2021 (Table S1).

When comparing the cases detected and reported in the diagnostic setting with those newly identified, differences in the fungal content and extent of infected tissue were revealed by the histopathological examinations. Whereas the previously reported IFIs showed in 61.5% high, in 23.1% moderate, and in 15.4% of cases low fungal content and amounts of infected tissue, the majority of the newly identified mycosis had moderate loads (46.4%), followed by low (28.6%) and high loads (25.0%). For the whole cohort with mycosis, 37.2% showed high, 37.2% moderate, and 25.6% low fungal loads (Fig. 1A). Figure 1B-G represents exemplary microscopic pictures of the three categories.

### Histological analyses of fungal infection pattern in liver transplants

**Fig. 3:**
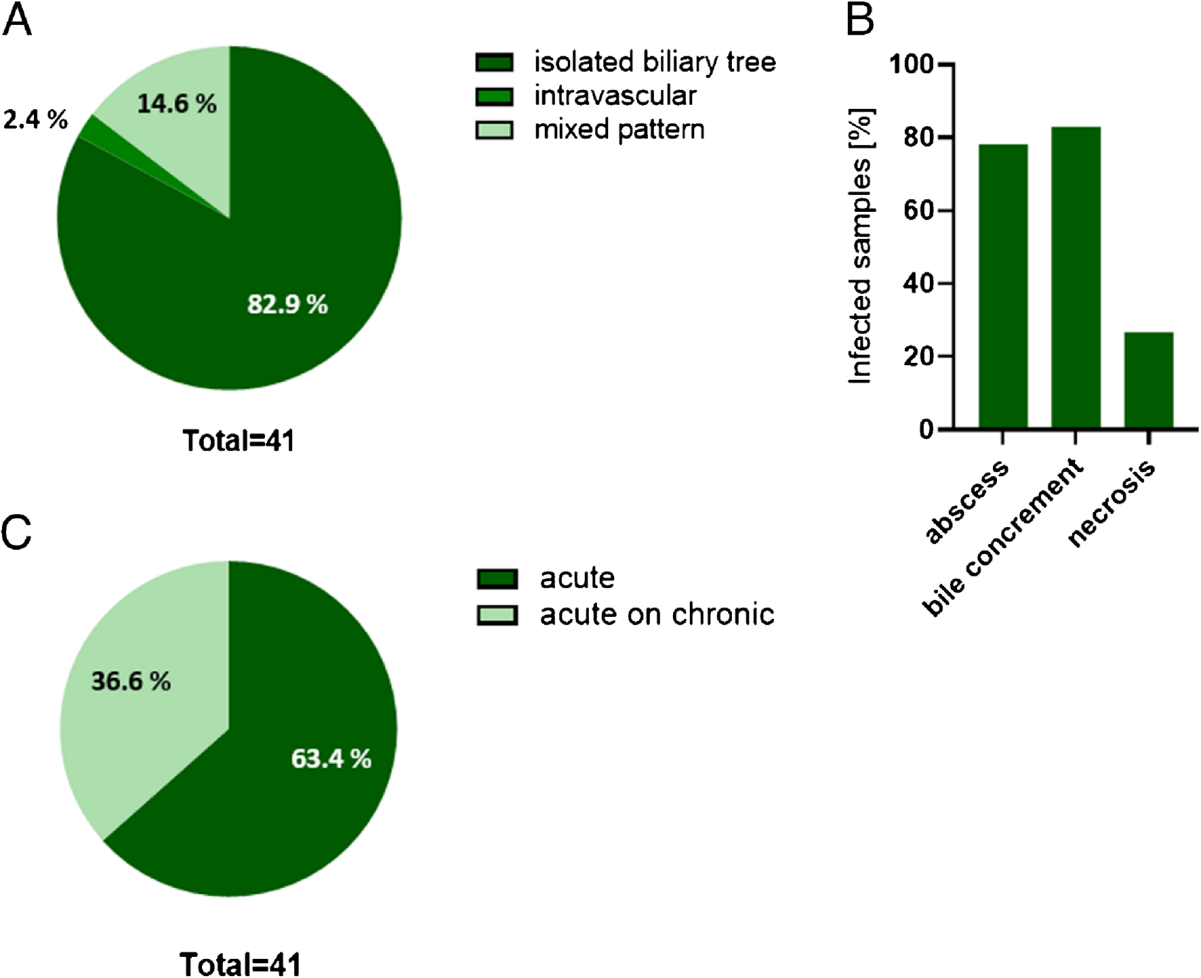
Morphological characterization of IFIs. The 41 identified cases of IFIs were investigated for histopathologic characterizations. **A** Localization of fungal infection within the liver was classified in biliary tree (dark green), intravascular mycosis (medium green), and a mixed infection of both structures (bright green). **B** Morphological changes were classified in abscess formation, formation of bile concrements, and necrosis. **C** Infection was classified as acute (dark green) and acute on chronic infection (bright green)

In order to describe the fungal infection pattern in the liver transplant samples, the localization of the infection was characterized. In total, 97.6% of the explanted livers with fungal infection showed affection of the biliary tree, mainly the large bile ducts; whereby for 14.6% of all cases, an additional involvement of blood vessels was found. In a single case, only blood vessel associated fungal infection was detected (Fig. 3A).

For more specific investigations of the disease pattern, morphological changes connected with fungal affection were investigated. In 82.9% of cases, bile concrements were topographically associated with the fungal species, in 78.0%, abscess formation was present, and 26.8% of mycotic liver transplants showed significant tissue necrosis (Fig. 3B). In 63.4% of cases, only signs of acute inflammation were found, and 36.6% presented signs of acute and chronic inflammation (Fig. 3C). Figure S2 exemplarily shows the morphologic pattern found in the histopathological investigations.

### Molecular identification of fungal species

**Fig. 4:**
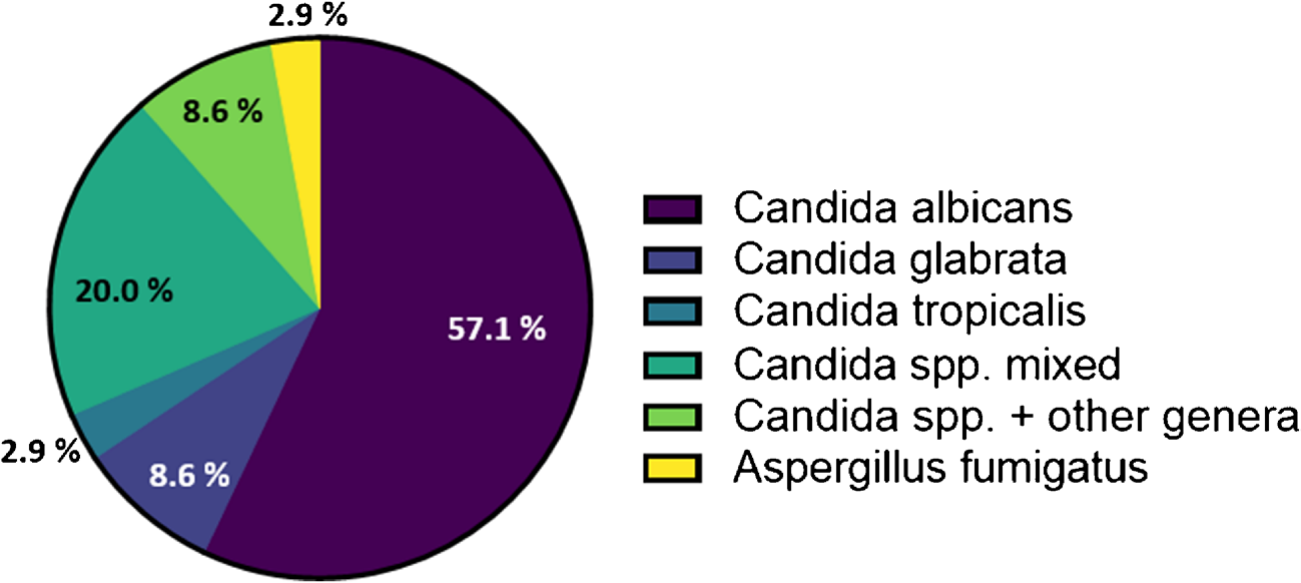
Pathogen pattern of IFIs on species level. From 35 samples, an identification of fungal pathogens on species level was performed

For characterization of the fungal species responsible for the affection of the liver transplants, further molecular pathological investigations were performed. In 35 of the 41 positive cases, the fungal species pattern could be determined by the assay employed. *Candida albicans* represented the most prevalent species, being present in 26 (74.3%) of cases. In 20 samples (57.1%), solely this species was found, whereas in the other six cases (17.1%), mixed infections with other *Candida* spp. were present. Five other *Candida* spp. were detected. *C. glabrata* was present in seven cases (20.0%), where it represented the only determined species in three samples (8.6%). *C. tropicalis* was present in one pure and two mixed infections. *C. krusei, C. lambica,* and *C. parapsilosis* were present in one mixed infection case each. Besides *Candida*, three other genera were identified. *Cryptococcus neoformans* and *Purpureocillium lilacinum* were present in one or two samples, respectively, as part of a mixed infection, whereas *Aspergillus fumigatus* was identified as sole infection in one case. All in all, in ten samples (28.6%), more than one pathogen was detected (Fig. 4, Table S2).

### Lethal mycotic infection of liver transplant patients

The cohort of this study included three transplant livers from autopsy cases (Table 2). In all cases, a septic multiorgan failure was the cause of death. In one case, a clear pathogenetic connection between the fungal species *C. glabrata*, identified in the transplant liver by molecular pathological investigations as well as clinically, could be drawn (Table 2, patient no. 1). For the other two cases, no fungal species could be molecularly identified in the autopsy liver tissue (Table 2, patient no. 2, 3).

**Table 2:** Characteristics of the investigated autopsy cases including previous explanted transplant livers

Patient number	Explanted liver (E) Autopsy liver (A)	Transplant survival (days)	Sample included in cohort	Presence of mycosis	Identified fungal species
1	A	126	+	+	*C. glabrata*
2	E	1756	+	-	-
	A	1989	+	-	-
3	E	4272	+	-	-
	A	343	+	-	-
4	E	648	+	+	-
	A	13	-	+	*C. glabrata*
5	E	1300	+	+	*C. glabrata, C. parapsilosis*
	A	33	-	+	*C. krusei*

Two retransplanted autopsy cases that were excluded from the cohort, as they did not meet the criterium “transplant survival > 90 days,” were also investigated for the presence of mycoses (Table 2, patient 4, 5). For these cases, septic multiorgan failure was also the cause of death. In both cases, a fungal recolonization within the short transplant survival time (13 and 33 days) took place, as *C. glabrata* or *C. krusei* were identified in the autopsy liver.

## Discussion

The aim of this comprehensive analysis of all explanted transplant livers between 1991 and 2021 at the University Hospital Heidelberg was to gain more insights into the prevalence, pathogenesis, and diagnostic and clinical relevance of IFIs in chronic liver transplant failure. The study cohort is a complete representation of liver transplant recipients with chronic transplant failure and posttransplant survival > 90 days at our site without further selection-bias. After reevaluation and comprehensive reexamination, a mycotic infection of the explanted transplant liver was identified in 27.5% of cases. Prevalence numbers in liver transplants given in the literature so far vary greatly between 2 and 42% [5, 6, 9, 10], but are unreliable, as they either report selected subcollectives, additionally extrahepatic mycoses, and/or lack comprehensive histopathological reevaluation of the explanted liver tissue. Of course, the percentage of positive cases in our study is likely to represent some underestimation, as it could only reassess the archived paraffin blocks. False negative cases, in which fungal infection was missed during grossing and selecting for histological examination, may exist. However, it is a reasonable estimate that fungal transplant liver infection represents a relevant contributor or worsening factor in roughly one-third of chronic liver transplant failures. There are numerous factors that may modulate this frequency, like therapeutic strategies in chronic transplant insufficiency and specifically antimycotic treatment. Furthermore, differences in the characteristics of patients and transplanted livers are likely to matter. In a retrospective study from Austria investigating liver explants from patients with transplant survival > 90 days (*n* = 197), IFI was diagnosed in 8.6% of cases. This was associated with an almost five times higher chance of death [10], emphasizing the dismal outcome of mycotic transplant infections. However, the IFI cases in this study did not specifically represent mycotic infection of the liver, but of all organs. Additionally, data were collected retrospectively and without histopathological reevaluation, which is likely to lead to significant underestimation of the mycotic infection burden. We could show an increase of mycotic transplant infections after the first observation decade. This seemingly paradox effect is likely to depend on improved management of other transplant complications, such as acute and chronic rejection and improvements in transplant surgery allowing for longer transplant survival [20] and a higher chance for fungal infection of the transplant. On the other hand, overall transplant survival was shorter in transplants with mycotic infection compared to those without, supporting the negative effect and clinical relevance of this complication. Additionally, the already clinically diagnosed cases had a longer transplant survival compared to the cases that were identified solely by our histopathological analyses (data not shown). This indicates that after identification and treatment, transplant outcome can be improved.

The comprehensive histopathological analyses give some clear insights into the pathogenesis of the fungal transplant infection. It is in its vast majority an infection of the biliary tree and not a hematogenous infection of the liver parenchyma. It primarily affects the large bile ducts and not the small peripheral ducts. It is obviously connected to florid, granulocytic infection, which frequently destructs the bile ducts, leading to cholangiogenic abscess formation and with time fibrosis induction. A clear feature is the frequent connection of mycotic infection and intrahepatic lithiasis, sometimes even showing fungal inclusion in or outgrowth from the concrements. Our analysis does not allow to distinguish whether fungal infection initiated concrement formation or whether existing concrements facilitated the fungal infection, e.g., via bile stasis, and provide a soil poorly accessible to antimycotic therapy [10]. All mechanisms may exist and are likely to form a vicious cycle worsening fungal infection, cholangitis, and transplant function.

By this, several conclusions regarding clinical management are suggested. Diagnosis and clinical management of mycotic transplant infection may largely concentrate on the status and function of the large bile duct and especially intrahepatic lithiasis, related abscess formation, and their prevention. The data suggest that significant bile duct destruction with concrement and abscess formation may represent a point of no return, from which on even dedicated antimycotic treatment may not be able to eliminate fungal infection. Therefore, preventive measures, early diagnosis, and treatment targeting intrahepatic lithiasis and bile stasis are likely to be relevant.

Several indications for the clinical relevance of fungal infections can be derived from our study. Firstly, the sheer number of infected cases combined with the obvious inability to eliminate the infection warrants intense attention not only regarding deterioration of the transplant liver, but also as potential source of life-threatening fungal sepsis. Secondly, the fact that transplants with proven fungal infection show shorter survival compared to those without proven infection is of major interest. Thirdly, the direct pathogenetic link to concrement and abscess formation establishes the need for intervention, as both are severe clinical conditions that may deteriorate transplant function as well as the patient’s general performance status. In addition, the potential of antimycotic drugs for adverse liver effects is well known. Fourthly, liver transplant mycotic infection threatens the success of retransplantation. In this context, it is worth to mention that we identified two cases where death due to septic multiorgan failure occurred shortly (13 days/33 days) after retransplantation and where we could show fungal infection in the transplant organ. As the autopsy rate is low, the rate of fungal reinfection after retransplantation is certainly underestimated.

We have analyzed the mycotic species relevant for liver transplant infection. Most recent technology allowed for a high analytical success rate, with few cases escaping molecular specification due to old age of the specimen or low fungal load. *Candida* spp., mainly *C. albicans*, were identified as the by far most prevalent species, which is in accordance with previous reports (68–93%), as well as the low prevalence of *Aspergillus* (1–9%) and *Cryptococcus* (0.5–5%) [5, 6, 9]. Solely *Purpureocillium lilacinum* has not been described as a fungal pathogen in liver transplants. A further species reported in previous studies, the drug-resistant *C. auris* [5, 8, 13, 14], was not detected in our collective. Recent investigations show a shift from *C. albicans* towards infections by non-albicans species like *C. glabrata* and *C. parapsilosis*, and concomitant higher mortality rates [7–9, 13]. In our collective, the latter was detected in one case and *C. glabrata* in seven cases.

In a high percentage of cases (68%), the mycotic transplant infection was not reflected in the original diagnostic report. There are different reasons for this underreporting. Most importantly, the relevance of the mycotic infection in chronic transplant failure and for the further course of the patient and the respective therapy may not be obvious to the reporting pathologists and may therefore not be thoroughly assessed and reported. So far, to our knowledge, no recommendations or standard reporting forms exist that integrate the aspects of fungal infections into grossing, histopathological assessment, and reporting of liver explants. Furthermore, fungal infection may be overlooked in the histopathological assessment, especially if adequate special stains are not performed. Indeed, in our study, the non-reported cases showed lower fungal load compared to those cases in which the fungal infection was reported. According to our findings and in order to prevent missing fungal infections in the explanted liver, large bile ducts must be prepared in a dedicated manner during grossing and specifically inspected for macroscopic signs of destruction, abscess formation, and concrements. Respective alterations must be dissected and processed for histological examination. Adequate special stains (e.g., PAS, methenamine silver) should be performed of the respective lesions to facilitate identification of fungal species. The inclusion of fungal infection once detected into the final diagnostic report should be mandatory. Including these parameters in instruction and training protocols, respective recommendations, and/or structured reporting forms appears to be beneficiary. Additionally, prospective studies that follow these recommendations may further help to determine the real burden of mycotic transplant infection and design improved interventional measures. In support to this, we demonstrated that the IFIs in our collective were not detected in advance by biopsy of the transplant liver, and by clinic-serological data, a fungal infection of the transplant liver was known or suspected only in 36.6% of cases. This underscores the relevance of a comprehensive and structured workup of the transplant livers regarding fungal infection as it may influence posttransplant management, in our setting in more than half of the patients.

Our study has several limitations. It is retrospective, and therefore, no reassessment of all procedures prior to histopathological analyses (grossing, tissue selection for histopathologic analyses) was possible. On the other side, it is the first study on fungal transplant infection on such are large collective by complete expert-driven reanalysis and reassessment, generating higher data quality. Nevertheless, as mentioned above, prospective analyses based on structured protocols and data acquisition are needed. Besides this point, the categorization of the mycotic infection in “low,” “moderate,” and “high” histologically detectable fungal load represents only an estimate of severity of the infection due to the retrospective character of the study, which restricted the analyses to the existing FFPE samples, differing in amount, localization, and prevalence between the cases. The study is of single center character, which on one hand guarantees higher data consistency but may also be affected by center-based peculiarities. However, transplant center certification, accreditation of the Institute of Pathology, and respective tight quality assurance measures ascertain high level adherence to standard and consented procedures. Nevertheless, standardized multicenter studies or registries would help to eliminate a potential center-based bias. Furthermore, the study covers a long time span of three decades, which have seen dramatic improvements in post-transplant treatment protocols, antimycotic therapy, and other interventional measures [20]. Thus, some of the conditions included in this study may not be valid anymore. Nevertheless, fungal infection has remained relevant throughout the whole study period with increasing infection numbers.

In summary, our comprehensive single center study documents the high relevance of fungal infection in chronic transplant failure, establishes its predominant pathogenesis, and further supports its clinical and diagnostic relevance. By this, specific diagnostic and clinical measures can be derived that may improve the recognition and treatment of fungal transplant infection. These findings and measures may be of further help also in other solid organ transplantations.

## Supplementary Information

Below is the link to the electronic supplementary material.

Supplementary file1 (DOCX 2427 KB)

## Data Availability

There are no further sequencing, patient or histological data that could be provided for further analyses.
