# Supplementary material for: High relevance of invasive fungal disease in chronic liver transplant failure: a comprehensive cross-sectional study
Source: Virchows Arch. 2025 Feb 18;487(2):413–22. doi: 10.1007/s00428-025-04050-4 (PMC12391138; doi:10.1007/s00428-025-04050-4)
Supplement: Supplementary file 1 — Supplementary file1 (DOCX 2427 KB) [file 428_2025_4050_MOESM1_ESM.docx]

**SUPPLEMENTARY DATA**

**
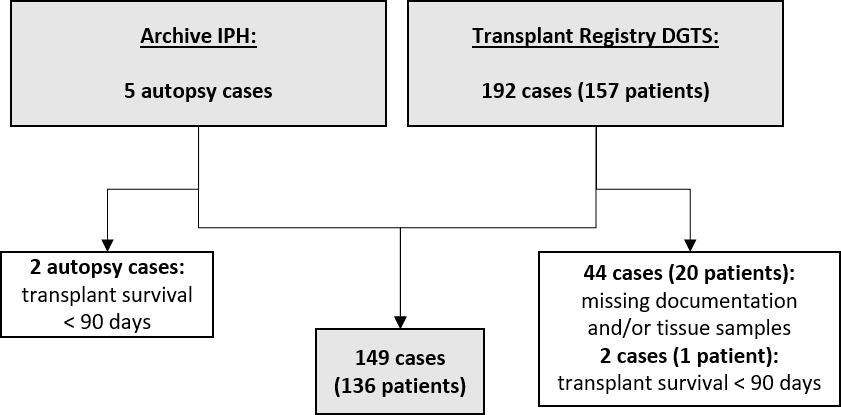
**

**Figure S1:** Recruitment of the case cohort

All 157 patients with explanted liver transplants representing 192 explanted transplant livers between 1991 and 2021 at University Hospital Heidelberg were filtered for patients with transplant survival ≥ 90 days and matched to tissue samples present in the Archive of the Institute of Pathology, Heidelberg (IPH). Patients not meeting the criteria as well as cases where no documentation or tissue samples were present were excluded from the cohort. Suitable autopsy cases from die Archive of the IPH were included. DGTS = Department of General and Transplant Surgery

**
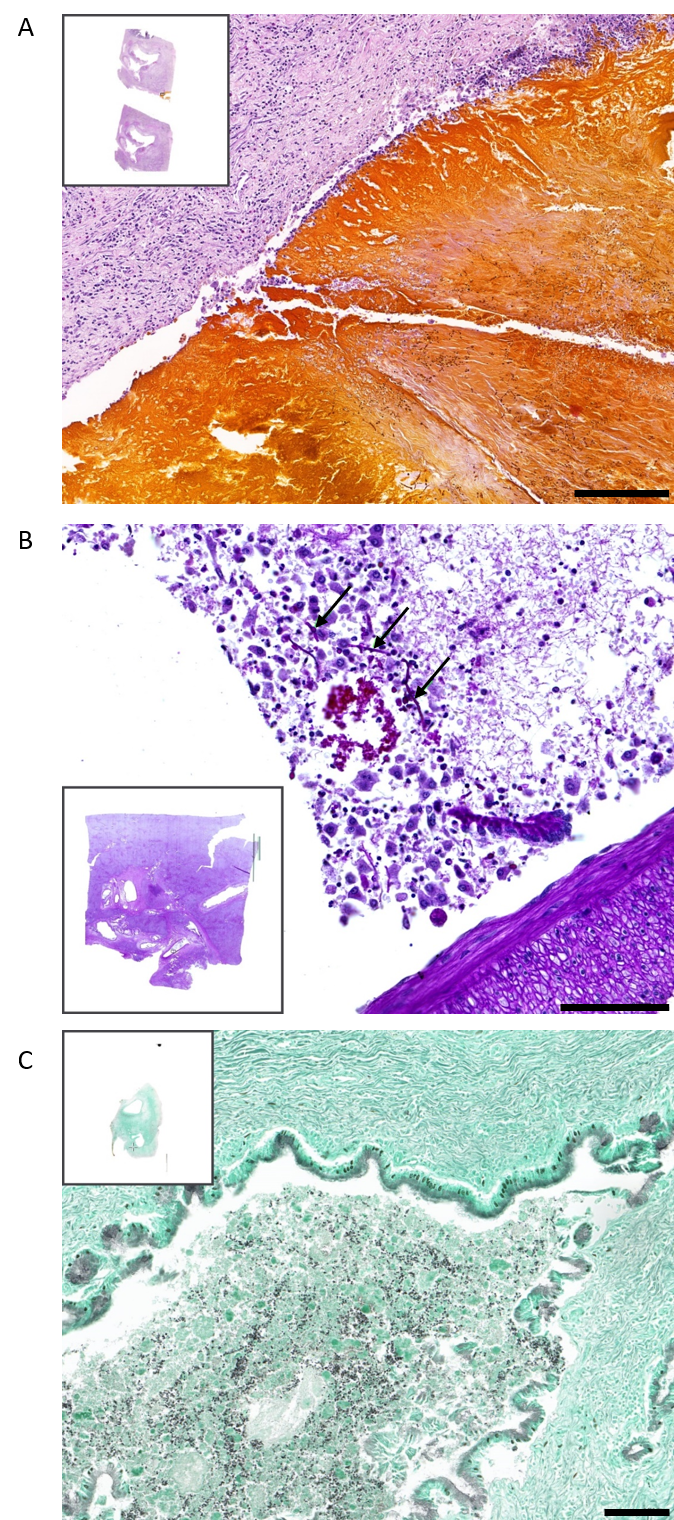
**

**Figure S2:** Exemplary histopathological findings in mycotic tissues

**(A)** Exemplary HE staining of a large obstructed bile duct with signs of acute cholangitis and fungal infection in the bile concrement. **(B)** Exemplary PAS staining of a portal vein branch with fibrin, solitary spores, and pseudohyphae (marked with arrows). **(C)** Exemplary GMS staining of a large bile duct with spores in the lumen and little inflammation. Scale bars: A = 200 µm; B, C = 100 µm.

**Table S1:** Number and percentage of liver explants with histopathologically identified mycosis between 1991 and 2021, presented for the year of liver implantation and explantation.

|  |  |  | year of implantation | | | |  | year of explantation | | | |
| --- | --- | --- | --- | --- | --- | --- | --- | --- | --- | --- | --- |
|  |  |  | non-affected | mycosis | total | % mycosis |  | non-affected | mycosis | total | % mycosis |
| 1990-2000 |  |  | 18 | 6 | 24 | 25.0 |  | 12 | 1 | 13 | 7.7 |
| 2001-2010 |  |  | 55 | 17 | 72 | 23.6 |  | 40 | 18 | 58 | 31.0 |
| 2011-2021 |  |  | 32 | 18 | 50 | 36.0 |  | 57 | 22 | 79 | 27.8 |

**Table S2:** Identified fungal species with the corresponding number of samples from which they were extracted in pure or as part of a mixed infection.

| Species | pure infection | mixed infection | total |
| --- | --- | --- | --- |
| *Candida albicans* | 20 | 6 | 26 |
| *Candida glabrata* | 3 | 4 | 7 |
| *Candida tropicalis* | 1 | 2 | 3 |
| *Candida krusei* | 0 | 1 | 1 |
| *Candida lambica* | 0 | 1 | 1 |
| *Candida parapsilosis* | 0 | 1 | 1 |
| *Purporeocillium lilacinum* | 0 | 2 | 2 |
| *Cryptococcus neoformans* | 0 | 1 | 1 |
| *Aspergillus fumigatus* | 1 | 0 | 1 |
